# Object Tracking Based on Vector Convolutional Network and Discriminant Correlation Filters

**DOI:** 10.3390/s19081818

**Published:** 2019-04-16

**Authors:** Yuan Liu, Xiubao Sui, Xiaodong Kuang, Chengwei Liu, Guohua Gu, Qian Chen

**Affiliations:** School of Electronic and Optical Engineering, Nanjing University of Science and Technology, Nanjing 210094, China; liu_yuan_eo@163.com (Y.L.); 113104000436@njust.edu.cn (X.K.); njust_liu@163.com (C.L.); gghnjust@163.com (G.G.); cq1964@163.com (Q.C.)

**Keywords:** object tracking, convolutional neural network, discriminant correlation filter

## Abstract

Due to the fast speed and high efficiency, discriminant correlation filter (DCF) has drawn great attention in online object tracking recently. However, with the improvement of performance, the costs are the increase in parameters and the decline of speed. In this paper, we propose a novel visual tracking algorithm, namely VDCFNet, and combine DCF with a vector convolutional network (VCNN). We replace one traditional convolutional filter with two novel vector convolutional filters in the convolutional stage of our network. This enables our model with few memories (only 59 KB) trained offline to learn the generic image features. In the online tracking stage, we propose a coarse-to-fine search strategy to solve drift problems under fast motion. Besides, we update model selectively to speed up and increase robustness. The experiments on OTB benchmarks demonstrate that our proposed VDCFNet can achieve a competitive performance while running over real-time speed.

## 1. Introduction

Visual object tracking is one of the fundamental problems in computer vision with numerous applications [1,2], such as cameras surveillance and human–computer interaction. A typical setting of object tracking is tracking an unknown object in a video sequence, while a single labeled target is provided as a bounding box in the first frame [3,4]. Several trackers design the feature models to represent targets and predict targets by searching for the image region to choose the most similar one. It proves that more accurate feature representations will lead to better tracking performance [5]. To match object appearance accurately, recent tracking algorithms focus on proposing effective representation schemes, such as points, texture [6], histograms, optical flow, or perceptual hashing [7]. However, object tracking is still a challenging problem since the object appearance tends to change and be disturbed by surroundings from frame to frame.

Recently, convolutional neural network (CNN) has been applied to various computer vision tasks such as image classification, object detection and so on. This attributes largely to CNN powerful feature representations. Several state-of-the-art CNN-based tracking methods [8,9,10,11,12,13] have been proposed in recent years. CNN-SVM [8] takes the feature maps of a pre-trained CNN as feature descriptors to learn discriminative target appearance models by using an online Support Vector Machine. CNT [9] uses a large amount of auxiliary data to train online a shallow convolutional network for visual tracking without offline training. For encoding target’s structural information, the auxiliary data is obtained by extracting patches from the target region as adaptive contextual filters. To take advantage of large-scale visual tracking data, MDNet [10] proposes to pre-train deep CNN in multi domains, where domains correspond to individual training sequences. In Ref. [11], Chi et al. exploits the hierarchical features in different layers of a deep model and design a dual structure to obtain better feature representation from various streams. Besides, some works [12,13] also utilize the Siamese network to learn template matching without online updating while possessing high tracking speed. GOTUON [12] employs the Siamese network and regresses directly to the location of the target object with no online training required. Furthermore, SiamFc [13] builds a fully-convolutional Siamese network to learn the similarity function of the two inputs. With the large online training samples and enormous parameters, these trackers have achieved great performance. However, keeping real-time speed and small memory are also vital for visual tracking as well as accuracy and robustness.

By solving a ridge regression problem efficiently in Fourier frequency domain, DCF-based trackers [14,15,16,17,18] have achieved a great balance between performance and speed recently. MOSSE [14] is a famous pioneering work that first employs DCF to object tracking. By exploiting the single-channel gray features, MOSSE can operate more than 600 FPS. CSK [15] adopts circulation matrices to fast learning and detection with the Fast Fourier Transform. In Stape [16], Bertinetto et al. combine a color statistics-based model to get complementary cues in a ridge regression framework. By learning DCF based on a scale pyramid representation, DSST [17] reaches accurate scale estimation. To deal with the tracking drift, Reference [18] proposes an adaptive updating strategy which uses cosine similarity together with two peak-to-sidelobe ratios and max response of scale tracker as confidence metrics for failure detection. Several recent works [19,20,21] have combined CNN with the fast shallow tracker based on correlation filters. HCF [19] extracts hierarchical convolutional features from the pre-trained VGGNet and puts the features into correlation filters to regress the response map. In order to take full advantage of features from different CNN layers, HDT [20] utilizes an adaptive Hedge method to hedge several weak trackers into a stronger one. In Reference [20], several weak trackers are constructed by using correlation filters where each one is trained with pre-trained VGGNet features from one layer. To learn the convolutional features and perform the correlation tracking process simultaneously, Discriminant Correlation Filters Network Tracker (DCFNet) [21] treats DCF as a special correlation filter layer added in a Siamese network to learn the convolutional features.

In this work, we choose DCFNet [21] as our baseline approach. Common DCF approaches use the features from a CNN pre-trained for a different task, leading to the problem that the features extracting process and the correlation tracking process are independent. The architecture of DCFNet [21] is similar to SiamFc [13], which builds a fully-convolutional Siamese network. Siamese network employs the same CNN to extract features of two image patches and combine their representations using a similarity metric. The difference between DCFNet [21] with SiamFc [13] is the similarity metric. DCFNet treats DCF as a special correlation filter layer to serve as the similarity metric and output a probability heatmap of target location. In the convolutional stage of DCFNet [21], Wang et al. set the network lightweight (75 KB) which consists of two convolutional layers without pooling layers. However, we observe that DCFNet [21] fixes the search region the same size as the exemplar region. It is probably limited by the special correlation filter layer where a Hadamard product is used between the features of search patch and correlation filter. This will lead to the problems that the exemplar region contains too much surrounding information or the search region is not large enough to search for a large displacement object. 

**Figure 1:**
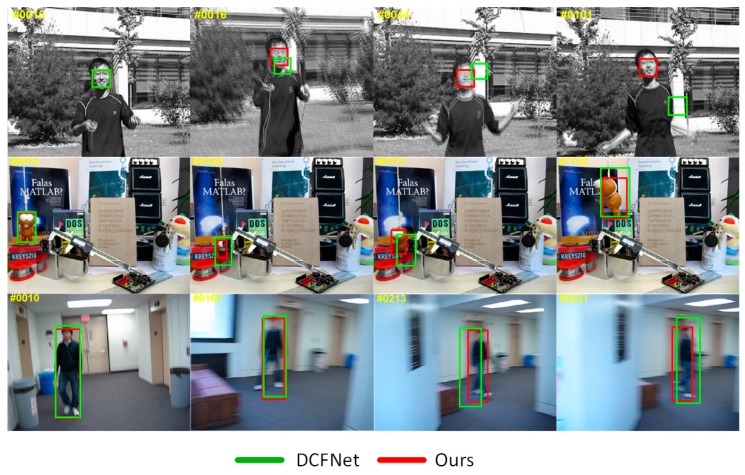
A comparison of our method with the baseline approach of DCFNet [21]. The attributes of three sequences correspond to fast motion (top row), occlusion and deformation (middle row), and motion blur (bottom row). Thanks to these strategies mentioned above, our tracker can track a target successfully.

In this paper, we propose a novel light-weighted tracking algorithm, namely VDCFNet, and combine VCNN with DCF. Similar to DCFNet [21], we also treat DCF as a correlation filter layer added in a Siamese convolutional network to learn objects’ similarity in Fourier frequency domain. The benefit of this is that we can train an end-to-end network for visual tracking. We introduce the VCNN to simplify the network and reduce parameters. Specifically, we replace one traditional convolutional filter with two novel vector convolutional filters in the convolutional stage of our network. In spite of fewer parameters and a slimmer model, we still take full advantage of the light network to extract objects’ representations. In the online tracking stage, we propose a coarse-to-fine search strategy, which can get a great balance between stability and accuracy to solve the fixed search region problem. By updating the model selectively, our tracker ignores the disturbance in short time and pays more attention to the long-term changes. All these mentioned strategies keep our tracker tracking targets robustly compared with DCFNet [21], as shown in Figure 1. These enable our tracker to run at the speed of about 50 FPS during tracking, which still achieves a competitive performance compared to other state-of-the-art trackers.

The main contributions of this paper are as follows:

1. We introduce the VCNN to reduce the parameter set of appearance representation.
2. A coarse-to-fine search strategy is proposed to solve drift problems under fast motion.
3. A robust online update strategy is introduced to ignore the low confidence disturbance.

The remainder of this paper is organized as follows: Section 2 gives the details of our proposed approach. Then, Experimental results are further analyzed in Section 3. Finally, Section 4 concludes this work.

## 2. Materials and Methods

In this section, we first demonstrate our overall method briefly. Secondly, we introduce the vector convolutional operator and present the architecture of our lightweight Siamese network. Then, we briefly formulate the discriminant correlation filter operation in the DCF layer. Afterward, the coarse-to-fine search strategy is illustrated. Finally, we show the online model update strategy.

### 2.1. Overview

In the convolutional stage of our network, we propose the VCNN to reduce the parameter set of appearance representation. In addition, we also treat DCF as a special correlation filter layer added in a Siamese network. In the online tracking stage, we propose a coarse-to-fine search strategy, which can get a great balance between stability and accuracy to solve drift problems under fast motion and motion blur.

**Figure 2:**
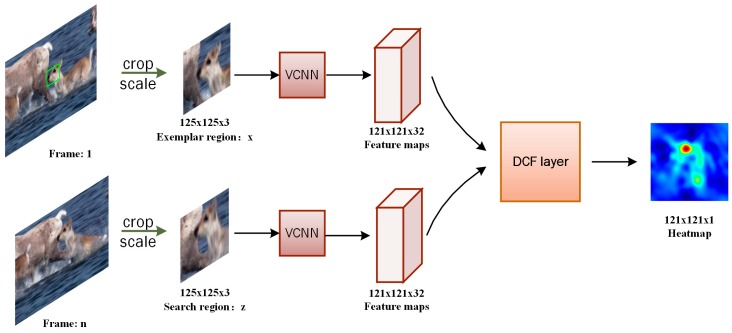
Our method architecture.

As a whole, we input into the Siamese network a search region from the current frame and an exemplar region from the first frame, and the network output the location and scale of the predicted target. To achieve this, we train a Siamese vector convolutional network purely offline with video sequences to compare the labeled target with the search regions. Two branches network share the same parameters and extract generic features to be compared with each other. The location of the target can be predicted by searching for the coordinate of the maximum value in the final probability heatmap, as shown in Figure 2.

### 2.2. Network Architecture

#### 2.2.1. Vector Convolution Operator

**Figure 3:**
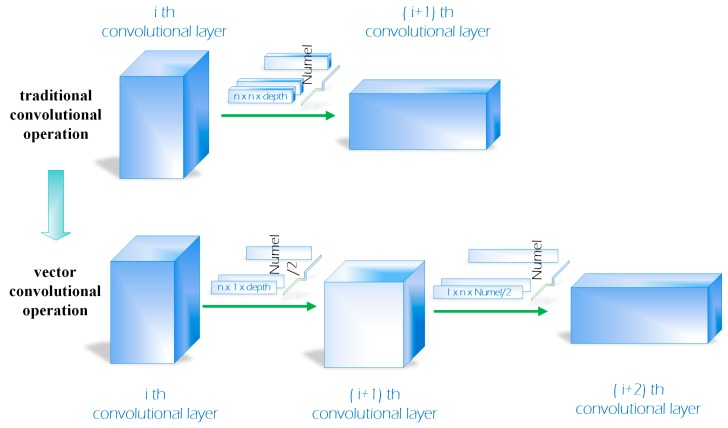
Our proposed vector convolutional operation.

We introduce a vector convolution operator in order to reduce parameters and save memory. To design a compact CNN for reducing parameters and computational costs, some works [22,23] have been proposed for object recognition. In Reference [22], separating 3×3 convolutions filters into two 1×1 convolutions filters is utilized in the Inception architecture. Replacing 3×3 convolutions with 1×1 convolutions was used in Reference [23] to create a very compact neural network and can achieve high accuracy with approximately 50× reduction of parameters. Our work is similar to these works as the reason that we compact the convolutional filters in the network. However, our compacted operator is the vector convolutional filters 1×n, n×1. Specifically, we separate every traditional convolutional filter into two vector convolutional filters in the convolutional stage of our network. As shown in Figure 3, a common convolutional filter denoted as numel@n×n×depth, is split into two vector convolutional filters which are denoted as (numel/2)@n×1×depth and numel@1×n×(numel/2), respectively. Although we add convolutional layers, our proposed vector convolutional operation saves approximately 20% memory compared with DCFNet [21] (75 KB). The larger the size of traditional convolutional filters become, the more arguments the vector convolutional operation will reduce. In spite of fewer parameters and a slimmer model, we still take full advantage of the light network to extract objects’ representations. The performance of our new convolutional model will be discussed further in the experimental section.

#### 2.2.2. Siamese Network

Our lightweight Siamese network contains two branches of CNNs, named as exemplar branch and searching branch. In this model, we input the labeled target region and search regions of the exemplar branch and searching branch, respectively, which extract the high-level representations from the two image patches. Since our purpose is to compare the search regions with the labeled object, we need to compute the similarity of the two features maps resulted from the two branches. To achieve this, a DCF layer receives the outputs of two branches to compute their correlation response map. Finally, the output of the Siamese network is a probability heatmap, which returns a high score if the two images depict the same object. Specifically, the convolutional stage in our tracking model consists of four vector convolutional layers. Besides, ReLU non-linearities only follow the second vector convolutional layer. We add an LRN layer at the end of the convolutional layers to restrict the magnitude of feature map values. More detailed network settings are given in Table 1.

#### 2.2.3. DCF Layer

We use DCF as a special layer like DCFNet [21]. In the standard discriminant correlation filters, we train a discriminative regression on the features of the object patch $\varphi (x)\in {\mathbb{R}}^{M\times N\times D}$, which has a Gaussian function label $y(m,n)=\exp(-({\left(m-M/2\right)}^{2}+{\left(n-N/2\right)}^{2})/2\sigma^{2})$. The correlation filter w can be obtained by solving the following minimization problem:

(1) $$w=\underset{w}{\arg\min}\sum_{m,n}{\left\|w•\varphi (x)-y(m,n)\right\|}^{2}+\lambda {\left\|w\right\|}^{2}$$

where • means circular correlation and the constant λ ≥ 0 is the regularization parameter. The minimization problem in Equation (1) can be solved in each feature channel by using fast Fourier transformation (FFT). Here, we let the capital letter Y denote the discrete Fourier transform F(y). The solution can be achieved as Ref. [14]:

(2) $$W^{d}=\frac{\varphi^{d}(x)⊙Y^{*}}{\sum_{k=1}^{D}\varphi^{k}(x)⊙{\left(\varphi^{k}(x)\right)}^{*}+\lambda }$$

In Equation (2), ∗ represents the conjugate operator and ⊙ denotes the Hadamard product. For a search region z in the new frame, the predicted target can be located by searching for the coordinate of the maximum value in the probability heatmap g, see Ref. [14] for more details.

(3) $$g={ℱ}^{-1}\left(\sum_{l=1}^{D}{\left(W^{d}\right)}^{*}⊙\varphi^{d}(z)\right)$$

Where ${ℱ}^{-1}$ denotes the inverse FFT transform. The objective function can be formulated as follows:

(4) $$\begin{array}{ll} & L(\theta )={\left\|g-y\right\|}^{2}+\gamma {\left\|\theta \right\|}^{2}\\ s.t. & g={ℱ}^{-1}\left(\sum_{d=1}^{D}{\left(W^{d}\right)}^{*}⊙\varphi^{d}(z,\;\theta )\right)\\ & W^{d}=\frac{\varphi^{d}(x,\;\theta )⊙Y^{*}}{\sum_{k=1}^{D}\varphi^{k}(x,\;\theta )⊙{\left(\varphi^{k}(x,\;\theta )\right)}^{*}+\lambda } \end{array}$$

According to Ref. [24], we can calculate the derivative per-element as follows since the operations in the forward pass only contain Hadamard product and division:

(5) $$\frac{\partial L}{\partial g_{\mathrm{uv}}^{*}}={\left(ℱ\left(\frac{\partial L}{\partial g}\right)\right)}_{\mathrm{uv}}$$

#### 2.2.4. Coarse-to-Fine Search

**Figure 4:**
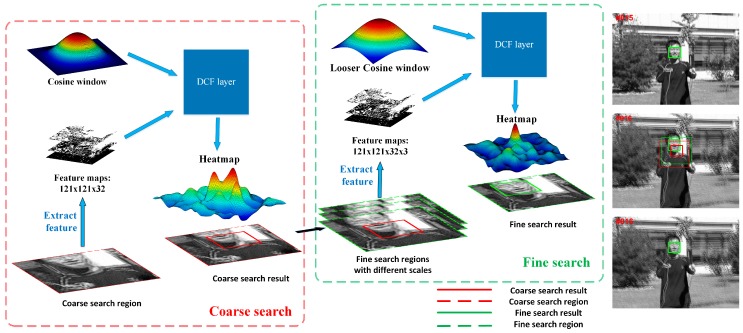
The overall coarse-to-fine search mechanism.

As we mentioned above, the search region has the same size as the exemplar region which is limited by the special correlation filter layer, leading to the problem that the search region is not big enough to search for a large displacement object. In order to adapt to the fast moving situation, our tracking model evaluates search regions K times in two manners, named as coarse search and fine search, as shown in Figure 4. In the coarse search stage, we only search for the object within one region centered at the previous target location and get a coarse predicted location. In the fine search stage, the scale and more precise location are achieved through evaluating regions centered at the coarse predicted location. The coarse-to-fine search can be considered as an iterative process that the accuracy tends to be better as the iterations increase. In practice, we found that searching twice can get a great trade-off between speed and accuracy. Note that the coarse-to-fine search is conducted in the same tracking model. Considering our light and slim model, the increase of time-consumption caused by the coarse-to-fine search can be ignored in a way.

In the coarse searching stage, we crop and scale two image patches, one is centered at the labeled object in the first frame and the other one is centered at the previous target in the current frame. The network output a probability heatmap in which the coordinate of the highest score locates the predicted target object. On the basis that the search region centers at the previous target, the displacement of the target will be obtained through multiplying the coordinate of the highest score by the entire stride of the network. In this stage, we only search for the object within one region of 2.5 times its previous size. In addition, a traditional cosine window, whose role comes from the periodicity assumption of the FFT, is used to learn the DCF filter.

After the coarse search, we assume that the tracking model finds the location near the real object, as shown in Figure 4. Then, our tracking model evaluates search regions centered at the coarse predicted location again. Besides, we change the traditional cosine window to a looser one. In practice, we discover that a looser cosine window can improve the tracking results and reduce the iterations of researching indirectly. In the fine search stage, multiple scales are evaluated at once by assembling a mini-batch of scale transformation regions.

#### 2.2.5. Online Model Update

Since the feature maps of our network are still large, maintaining a large new sample set is computationally expensive. After predicting the new target position, we re-crop the image patch center at the new target position from the current frame for updating the correlation filter. In practice, the optimization problem in Equation (2) can be formulated in an incremental mode as in Ref. [19].

(6) $$A_{t}^{d}=(1-\alpha )A_{t-1}^{d}+\alpha \cdot \varphi^{d}(x)⊙Y^{*}$$

(7) $$B_{t}^{d}=(1-\alpha )B_{t-1}^{d}+\alpha \cdot \sum_{k=1}^{D}\varphi^{k}(x)⊙{\left(\varphi^{k}(x)\right)}^{*}$$

(8) $$W_{t}^{d}=\frac{A_{t}^{d}}{B_{t}^{d}+\lambda }$$

where the parameter α ≥ 0 is the learning rate and t is the frame index. The advantage of this incremental update is that we do not have to sample the online training set to update our whole network.

**Figure 5:**
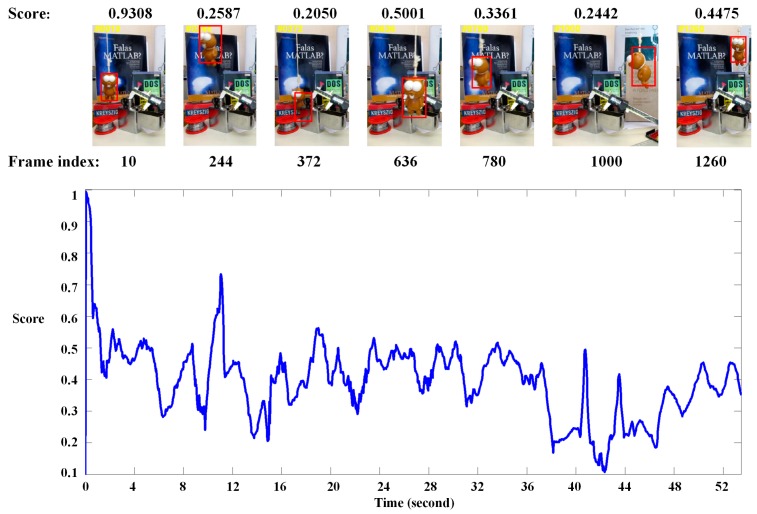
Our tracking results on a typical sequence. The y-axis indicates the maximum value in the heatmap. The tracking results are reliable most of the time. Occasionally, e.g., frame #372 and frame #1000, the scores become low because occlusion and deformation situations occur.

As we mentioned above, the value in the heatmap denotes the similarity score between the label target and region patch in a new frame. If tracking failure or disturbance situation occur, the score will be lower, as shown in Figure 5. In this work, we regard the maximum value in the heatmap as a confidence score of the predicted target. Given the tracking result in frame n, if the score is lower than the threshold, our tacker will treat it as a disturbance by the unexpected situation, e.g., illumination variation, occlusion, deformation and so on. In this case, the tracking result will not update our model.

## 3. Results and Discussion

In this section, we evaluate our VDCFNet with some state-of-the-art tracking algorithms on OTB [3,4]. Then, we further analyze the contribution of each component in our system. The results demonstrate that our VDCFNet can achieve a competitive performance with other state-of-the-art trackers.

### 3.1. Implementation Details

Training image pairs are obtained by cropping exemplar and search patches that are centered on the objects in annotated videos. The images are chosen from two frames of a video sequence within the near frames. In detail, we choose training videos from three datasets, including NUS-PRO [25], TempleColor128 [26] and UAV123 [27]. Especially, we remove the sequences that overlap with the test set and the parts beyond 500 frames in every video, leading to 114,672 frames. For each video, we choose each pair of frames at least 15 frames apart and within 60 frames. We crop the image pairs of 1.5× padding size in order to allow the network to receive some contextual information surrounding the object. Then, we resized the cropped patches to 125 × 125 × 3 to the network. During the training stage, we apply SGD with a momentum of 0.9, fix the weight decay γ to 0.0005, and set the learning rate to 1×10^−5^. The network is trained for 50 epochs with a mini-batch size of 16, leading to a 59 KB tracking model. In the updating model stage, we fix the online learning rate α to 0.002 and set the update selection threshold to one-third of the maximum score in the second frame. The regularization coefficient λ is set to 1 × 10^−4^ and the Gaussian spatial bandwidth is set to 0.1. To handle scale variations, we use a patch pyramid with scale factors of $1.03^{\left\{-1,0,1\right\}}$ to search for the object over three scales. At the same time, we penalize scale changing with a factor of 0.98 to provide damping. During testing time, we set the searching iteration K to 2 in the coarse-to-fine searching stage. In other words, our tracker coarse searches once and the fine searches once.

The proposed VDCFNet is implemented in MATLAB 2014b with MatConvNet [28]. All experiments are conducted on a machine with Intel Core i5-3470 at 3.2 GHz, 16GB RAM and a single NVIDIA GeForce GTX 1080 GPU. Our online tracking method operates at 50 frames-per-second when searching three scales. The source code is available on https://github.com/WalkWander/VDCFNet.

### 3.2. Experiment Analysis

#### 3.2.1. Dataset

The OTB [4] is a popular benchmark for visual tracking which contains 100 tracking sequences. One-hundred tracking videos are defined as bounding box annotations with 11 different attributes, including illumination variation (IV), scale variation (SV), occlusion (OCC), deformation (DEF), motion blur (MB), fast motion (FM), inplane rotation (IPR), out-of-plane rotation (OPR), out-of-view (OV), background clutters (BC), and low resolution (LR). We follow the protocol in References [3,4] and report the results based on success plots and precision plots for evaluation.

#### 3.2.2. Comparison on OTB

To evaluate our proposed method performance, we compare our proposed VDCFNet with another eight state-of-the-art tracking algorithms, including CNN-SVM [8], SiamFc [13], CSK [15], Staple [16], DSST [17], HDT [20], DCFNet [21], and Struck [29]. Especially, compared with the baseline method DCFNet [21], the results would show the advantages and disadvantages of our algorithm. As the framework in our algorithm is a Siamese network, we choose the most famous Siamese network-based method like SiamFc [13]. Besides, we also select some popular DCF-based methods like CSK [15], Staple [16], DSST [17], and HDT [20] for evaluation. 

**Figure 6:**
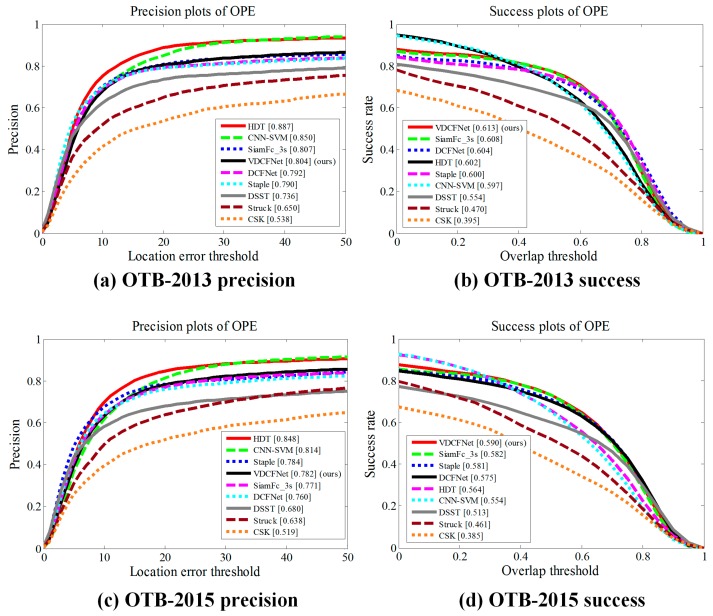
Distance precision and overlap success plots for OPE (one pass evaluation) of OTB2013 [3] and OTB2015 [4] benchmarks. The numbers in the legend indicate the representative precisions at 20 pixels for precision plots and the area-under-curve scores for success plots.

Figure 6 shows the results of VDCFNet in the metric of OPE on OTB2013 [3] and OTB2015 [4]. In particular, SiamFc_3s [13] denotes the SiamFc [13] method and evaluates three scales. In Figure 6a,c, the numbers in the legend denote the representative precisions at 20 pixels. In the case that the target may be too small relative to 20 pixels, we suggest readers pay more attention to the success plots and the overall performance of precision plots in Figure 6. Our proposed method with fewer parameters (59 KB) performs favorably against other state-of-the-art methods in the metric of OPE on OTB2013 and OTB2015 benchmarks. Our tracking model leads to 7.7% gains in success plots on OTB2015 compared with DSST using HOG features. With the more robust search strategy and online update strategy, the proposed method performs better compared to the recent DCFNet [21]. In Figure 6d, our VDCFNet tracker can achieve a significant improvement in OPE.

**Figure 7:**
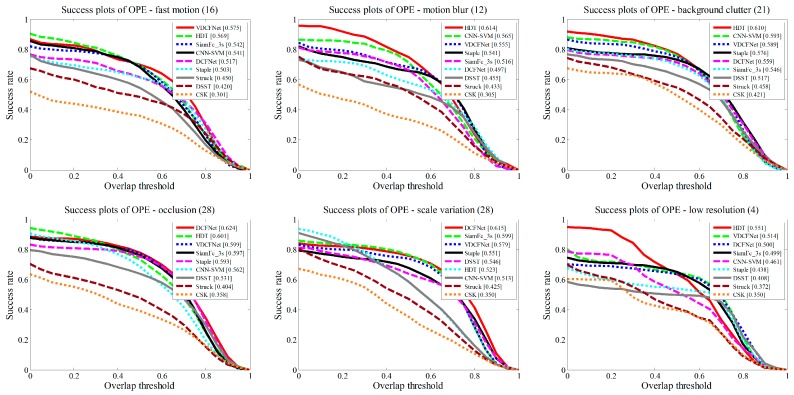
The success plots for six challenge attributes, including fast motion, motion blur, background clutter, occlusion, scale variation, and low resolution.

To further analyze the trackers’ performance, we also report the results on different challenge attributes on OTB2013, such as fast motion, occlusion, motion blur, etc. Figure 7 demonstrates the OPE for six main video attributes. In addition, Table 2 shows the results of all 11 attributes. Compared with the other trackers, our approach achieves smooth performance on all attributes. It is probably the reason that our tracker performs competitively on the overall score. In particular, our method is effective in handling fast motions. Compared with DCFNet [21], our method leads to 11.6% gains in motion blur attribute and 11.2% gains in fast motion attribute.

#### 3.2.3. Ablative Analysis

We also conduct the ablative analysis on OTB2013 [3]. In Table 3, the configuration of ablative analysis is listed in detail. We implement and evaluate several variations of our VCNN architecture with the same hyper-parameters in both training and testing stages. The effectiveness of our proposed VCNN is examined by comparison with the different vconv layers (vconv1-dcf, vconv2-dcf, vconv3-dcf and vconv5-dcf), where vconv1 denotes 2 VCNN layers, vconv2 denotes 4 VCNN layers, vconv3 denotes 6 VCNN layers, and vconv5 denotes 10 VCNN layers; all the vconv filters are 3 × 1, 1 × 3 with 16 or 32 numels, and dcf denotes the DCF layer. To verify objectively, we also test the normal same convolutional layers architectures (conv1-dcf, conv2-dcf, conv3-dcf, and conv5-dcf), where all the conv filters are 3 × 3 with 32 numels. Unlike image classification, the performance decreases with the layers going deeper. We partly attribute this effect to the decreased spatial resolution in the deeper layers. Intuitively, better spatial resolution is more helpful for locating the target accurately, which is crucial for the tracking problem. It is also probably that our training set is not enough for training the deeper network. Regarding the process speed, although we add more layers to the network, the VCNN constructions still perform competitively with the normal convolutional network. This is mainly because there is no need for backpropagation which costs most of the computing time in our tracking stage. In terms of parameter numbers, compared with conv3-dcf, the vconv3-dcf utilizes VCNN construction to gain a 58.8% smaller quantity of parameters and achieve even better performance under the CLE metric.

**Figure 8:**
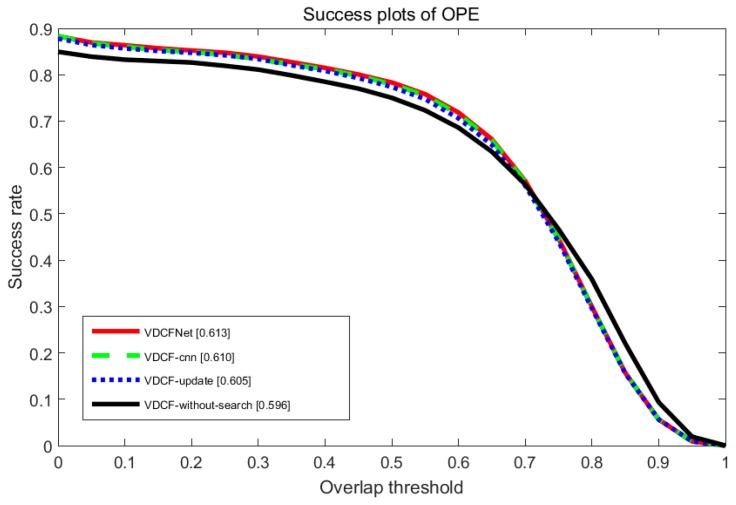
Performance evaluation of our proposed method without each part on OTB2013.

To evaluate the contribution of each part in our algorithm, we also investigate three additional versions of our tracking algorithm—VDCFNet without update selection and finetune model every frame (VDCF-update), VDCFNet without coarse-to-fine search (VDCF-without-search), and VDCFNet with normal convolutional operation (VDCF-cnn). Our entire algorithm performs better than all the variations. In addition, each component in our algorithm is beneficial to improve performance. The detailed results are illustrated in Figure 8.

#### 3.2.4. Qualitative Evaluation

**Figure 9:**
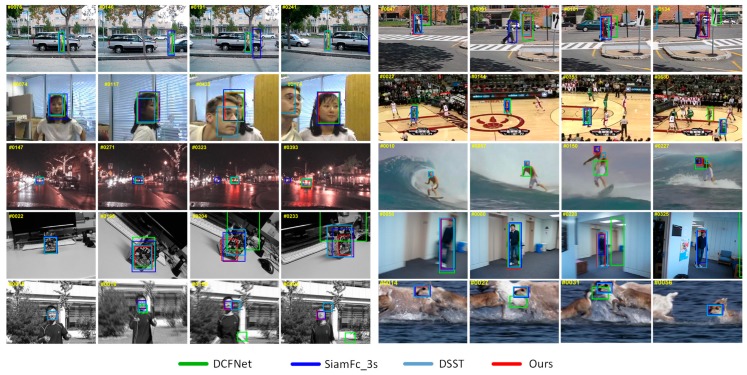
Qualitative evaluation of proposed algorithm on 10 challenging sequences from OTB100 (from left to right and top to down are Divid3, Couple, Girl, Basketball, Cardark, Surfer, Vase, Blurbody, Jumping, and Deer, respectively). The yellow number in the upper left corner indicates the frame index.

Figure 9 shows the tracking results of some state-of-the-art trackers: SiamFc_3s [13], DSST [17], DCFNet [21], and the proposed method VDCFNet on 10 challenging sequences from OTB100. In terms of fast motion, our tracker performs better than DCFNet [21] in Couple, Jumping and Deer sequences. In practice, our tracker fails to search for the target at frame #91 in Couple sequence. This is due to the large displacement of the target caused by the fast motion of the camera. Despite this failure, our tracker search back soon as show at frame #101 in Couple sequence. This may owe largely to our coarse-to-fine search strategy because search twice expands the search region.

For the Blurbody sequence, when heavy motion blur occurs, the DCFNet method fails to follow the target at frame #228 as it is disturbed by updating mode every frame. Especially the tracking model receives the bad update information when the tracking results drift. It becomes worse and worse as shown at frame #13 and #304 in the Jumping sequence. Only SiamFc [13] method and ours were able to track successfully. Compared with SiamFc [13] using the huge training dataset and deeper network, our method performs well with a shallow network.

In terms of in-plane rotation and scale variation, our algorithm performs better than the other three methods in the Vase sequence. Regarding the occlusion, SiamFc [13] and DSST [17] fail to track the object in Divid3, Girl and Basketball sequences. For the Divid3 sequence, the object occludes by a tree at frame #191. SiamFc [13] lost the target and stayed in the previous position. It is probably limited by the cosine window added at the response map in SiamFc [13] which increases the center weight and decreases the boundary weight. Benefitting from the property of DCF module and a large search region, our proposed tracker was able to overcome the disturbance caused by occlusion.

#### 3.2.5. Failure Analysis

**Figure 10:**
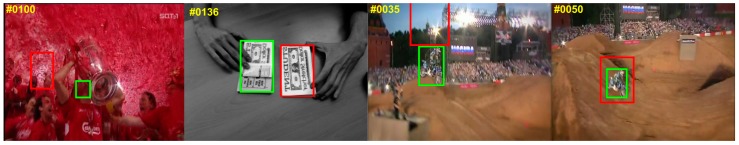
Failure cases on sequences from OTB100 (from left to right are Soccer, Coupon, MotorRolling, and MotorRolling, respectively). The red bounding box is our proposed method results and the green one indicates the ground truth. The yellow number at the upper left corner indicates the frame index.

However, we also discover a few failure cases as shown in Figure 10. For the Soccer sequence, when there is heavy background clutter and long-term occlusions occur, it is a really challenging job for our proposed tracker. Regarding the Coupon sequence, it is also difficult to distinguish the similar object for our method. Besides, we discover that our tracker is not sensitive to large rotation variation in some failure results such as MotorRolling sequence. Although our method is able to track the target back later at frame #50 which is owed largely to the coarse-to-fine search strategy, our tracker cannot handle the scale variation well after these failures in MotorRolling sequence. It is probably that our training set does not contain enough image pairs with the attribute of rotation variation. We will study these issues in the future.

## 4. Conclusions

In this work, we have proposed a novel tracking algorithm combined with the vector convolutional network with discriminant correlation filters. In the convolutional stage, we propose novel vector convolutional filter to simplify the network and reduce parameters. In the online tracking stage, we propose a coarse-to-fine search strategy to solve drift problems under fast motion. In the online update model stage, a robust online update strategy is introduced to ignore the low confidence disturbance. These enable our tracker to follow the target robustly with fewer parameters. The experiments have shown that our proposed method can achieve a competitive performance while operating over real-time speed.

## Figures and Tables

**Table 1:** The architecture of Siamese network. Each network branch consists of four vector convolutional layers (vconv). The output of each layer is denoted as ‘channel@size×size’, where channel means the number of feature maps. The convolutional filters are denoted as ‘numel@width×height×depth’, where numel means the number of convolutional filters and width,height,depth means the size of convolutional filters.

Layer	Convolutional Filter	Stride	Exemplar Branch	Searching Branch
**Input**			3 @ 125 × 125	3 @ 125 × 125
**vconv1**	32 @ 3 × 1 × 3	1	32 @ 123 × 125	32 @ 123 × 125
**vconv2**	64 @ 1 × 3 × 32	1	64 @ 123 × 123	64 @ 123 × 123
**Relu**				
**vconv3**	32 @ 3 × 1 × 64	1	32 @ 121 × 123	32 @ 121 × 123
**vconv4**	32 @ 1 × 3 × 32	1	32 @ 121 × 121	32 @ 121 × 121

**Table 2:** The score of success plot on OTB2013 [3]. The bold fonts indicate the best performance.

Name	IV	SV	OCC	DEF	MB	FM	IPR	OPR	OV	BC	LR	Overall Score
Ours	0.558	0.579	0.595	0.573	0.555	**0.575**	0.557	0.573	0.578	0.574	0.514	**0.613**
SiamFc_3s	0.532	0.599	0.597	0.541	0.516	0.542	0.570	**0.589**	0.635	0.546	0.499	0.608
DCFNet	**0.574**	**0.615**	**0.624**	0.558	0.497	0.517	0.569	**0.589**	**0.668**	0.559	0.500	0.604
HDT	0.553	0.523	0.601	0.627	**0.614**	0.569	0.578	0.583	0.569	**0.610**	**0.551**	0.602
CNN-SVM	0.555	0.513	0.562	**0.640**	0.565	0.541	0.570	0.581	0.571	0.593	0.461	0.574
Staple	0.568	0.551	0.593	0.618	0.541	0.503	**0.580**	0.575	0.547	0.576	0.438	0.600
DSST	0.561	0.546	0.531	0.506	0.455	0.420	0.563	0.535	0.462	0.517	0.408	0.554
Struck	0.418	0.425	0.404	0.393	0.433	0.450	0.436	0.425	0.459	0.458	0.372	0.470
CSK	0.361	0.350	0.358	0.343	0.305	0.301	0.394	0.381	0.349	0.421	0.350	0.395

**Table 3:** Ablation study of our method VDCFNet with different VCNN architecture compared with normal CNN architecture on OTB2013 using mean overlap precision (OP) at the threshold of 0.5, mean distance precision (DP) at 20 pixels, mean center location error (CLE), mean speed, and quantity of parameters.

Tracker	OP	DP	CLE	FPS	Parameters
vconv1-dcf	0.88	0.84	11.56	65.04	1728
conv1-dcf	0.89	0.84	11.52	65.94	896
vconv2-dcf	0.86	0.80	13.06	53.62	4848
conv2-dcf	0.87	0.79	12.98	54.46	10144
vconv3-dcf	0.81	0.72	14.02	41.04	7968
conv3-dcf	0.82	0.76	14.38	43.26	19392
vconv5-dcf	0.57	0.51	17.68	33.12	14208
conv5-dcf	0.60	0.57	16.76	30.84	37888
